# A Bibliometric and Trend Analysis on the Water-Related Risk Assessment Studies for *Cryptosporidium *Pathogen

**Published:** 2015

**Authors:** Alireza MESDAGHINIA, Masuod YOUNESIAN, Simin NASSERI, Ramin NABIZADEH NODEHI, Mahdi HADI

**Affiliations:** 1 *Center for Water Quality Research (CWQR), Institute for Environmental Research (IER), Tehran University of Medical Sciences, Tehran, Iran*; 2 *Department of Environmental Health, School of Public Health, Tehran University of Medical Sciences, Tehran, Iran*; 3 *Center for Air Pollution Research (CAPR), Institute for Environmental Research (IER), Tehran University of Medical Sciences, Tehran, Iran*

**Keywords:** Bibliometric analysis, *Cryptosporidium*, Risk assessment, Scopus, Water

## Abstract

***Background:*** The bibliometric methods have been used in many disciplines of sciences to study the scientific production and research trends. In this study, they were used to investigate research trends related to the risk assessment of *Cryptosporidium *pathogen in water field.

**Methods**: Data were obtained on the Scopus database from 1993 to 2013. Research tendency was investigated by analyzing the distribution of languages, countries, journals, author keywords, authorship pattern and co-authorship relations.

**Results**: The English language was dominant language of all publications (96.36%). Number of articles in this field increased from 2 in 1993 to 29 papers in 2007 and then received to 19 at the end of 2013. United States produced 35.41% of all pertinent articles followed by United Kingdom with 10.76% and Australia with 9.92%. *Water Research Journal* published the most papers in this field, taking 11.62% of all, followed by *Journal of Water and Health* (10.92%) and *Water Science and Technology* (10.21%). The most productive authors were *Ashbolt NJ* form Canada that accounts about 1.51% of the total publications followed by *Rose JB *and *Haas CN *from United States. Authorship pattern analysis results show that literature does follow Lotka’s law (*P*=0.627).

**Conclusion**: A downward trend in the number of publications is likely to occur in future. The results of this bibliometric analysis may help relevant researchers realize the scope of the microbial risk assessment research of *Cryptosporidium*, and establish the further research direction.

## Introduction

Microbiological quality of drinking water is a major concern for water consumers, water suppliers, and public health officials. “The drinking water can transport the microbial pathogens to great numbers of people, and can cause subsequent illnesses” (1). The protozoan pathogens are a group of microbial pathogens those can contaminate drinking water (2). *Cryptosporidium *sp. is member of protozoan pathogens. Morbidity and mortality associated with protozoan parasite are high with more than 58 million cases of childhood diarrhea caused by protozoans per year (3). *Cryptosporidium *spp. have been recognized as pathogenic protozoans and waterborne pathogens (4-8), which are widely distributed in the environment and can contaminate drinking water with their oocysts. Many animals, including poultry and livestock, have been known as the sources of infection (9-11). Indeed, the spread of the pathogens in the environment is dependent on water bodies for their transmission or as a habitat for intermediate or final hosts (12). 

The parasite was initially described from the gastric glands of mice and found as a new species in 1912 (13).The Cryptosporidium is a main cause of diarrhea worldwide (14). Globally, emission of 3 × 10^17^ oocysts per year from humans and animals to surface water was estimated for Cryptosporidium (15). An estimated 1.4%–10.4% of all diarrhea episodes in china is attributed to *Cryptosporidium *(16). The infection risk of Cryptosporidium varied from 0.15% to 0.29% for adults and from 0.04% to 0.08% for children in public drinking water delivered by surface water systems in Sao Paulo State, Brazil (17). In China from 50 source water samples examined in Shanghai, 32% were positive for *Cryptosporidium *by EPA Method 1623 (18). The‌ infection risk of 10^− 4^ (1:10,000) is suggested as tolerable risk by EPA for a yearly exposure to microbial contaminations. The Outbreaks of infection with *Cryptosporidium *species involving from hundreds to many thousands of people have been reported in many developed countries such as United States and United Kingdom (19, 20). 

Molecular epidemiological studies have also documented the presence of *C. parvum *and *C. hominis* as the main species of *Cryptosporidium *spp. in humans in Mideast countries (21). The work of Ghalebin et al. (22) on Ardabil’s (a city in north west Iran) river water samples, showed that among 30 examined samples, 11 samples were positive for the presence of *Cryptosporidium *spp. This means the *Cryptosporidium *prevalence ratio in surface water is 36.6%. The most outbreaks of *Cryptosporidium *with waterborne transmission in Iran, is reported in Chaharmahal-va-Bakhtiari Province (23). They characterized *Cryptosporidium *spp. in water samples collected from recreational ponds. Out of thirty samples examined, 6 (20%) were positive for different Cryptosporidium spp. Restriction pattern analysis showed that C. parvum was the most prevalent genotype, followed by C. hominis and C. canis, respectively. The infection prevalence in children with diarrhea in Tehran, the Capital of Iran, and Qazvin, a city in the northwest of Tehran has been reported 2.4% and 2.55% respectively (24).The use of wastewater for irrigation of vegetables farms in Tehran is reported (25) as one of risk factors leads to the contamination of vegetables with a prevalence of 33% and provides a route by which *Cryptosporidium *can be transmitted to humans. Thus, the Cryptosporidial infections through water contamination are one of the major concerns in Iran and many counties.

Considering the global distribution of cryptosporidiosis, it may be a notifiable disease all over the world especially in developing countries. Given these, implementation of risk assessment methods for quantifying the infection risk of this pathogen from exposure to water (drinking water, reclaimed water, swimming water, agricultural water and industrial water, surface water and groundwater) may be inevitable. The infection risk from exposure to the *Cryptosporidium *can be quantified by risk assessment methods. The Quantitative Microbial Risk Assessment (QMRA) has been successfully applied to figure out relative public health risks in many developed countries (26). Familiarization with QMRA tool in developing countries may be provided a better strategy for controlling the disease. Therefore, it is necessary to provide a basis for better understanding the global development of research related to the assessment of *Cryptosporidium *water-related risks. The historical research review based on bibliometric analysis could help further understanding of this field of study.

The bibliometric methods have been used commonly in many disciplines of science and engineering to study the scientific production and research trends (27-30). In this study, a bibliometric method was used to investigate research trends related to the risk assessment of *Cryptosporidium *pathogen in water field. The research productivity data published in all Scopus journals from 1993 to 2013 is presented regarding the contribution of major regions of the world. The results could be the basis for a better understanding of the global development of research related to the risk of *Cryptosporidium *from water route and may lead to greater attention to the topics of microbial risk assessment especially in developing countries.

## Methods

The data were based on Scopus bibliographic database. Scopus offers about 20% more coverage than Web of Science, whereas Google Scholar offers results of inconsistent accuracy (31). For bibliometric analysis, the Scopus was searched with keywords “risk assessment” OR “risk management” OR “quantitative microbial risk” OR “QMRA” to search related information in author keywords and “water” AND “*Cryptosporidium*” keywords to search within the title, abstract, author keywords, to compile a bibliography of all publication related to the research on the risk of *Cryptosporidium *in water field. The final number of publications was 364.The collaboration type was determined by the addresses of authors, where the term SC was assigned if all researchers’ addresses were from the same country and the term MC was designated to those articles, which were coauthored by researchers from multiple countries (32, 33). All the following analyses referring to document type, language, journal, country and author were analyzed by R programming language v.3.0.2 (34). 

## Results

Altogether 364 publications met the selection criteria mentioned above, containing 4 document types. Journal articles (JOUR) was the most frequently used document type (302; 96.36%). Others were Serial publication (SER) (47; 12.91%), Conference proceeding (CONF) (14; 3.84%) and In Press publications (INPR) (1; 0.002%). Because the journal articles, which are peer-reviewed within this field, were dominant in the document types, only the journal articles were selected for further analysis and all others were discarded. 

***Distribution of publication outputs***

***Distribution of Languages and publication year***

The analysis of languages distribution revealed that English was the predominant language. Out of the 302 records retrieved up to February 2014, English occupies the first position with 291 article records (96.36%). This may be partly due to the fact that United States and United Kingdom were countries those published the most publications in this field and it is official language in many countries and many articles are published in English language only (35). There were five languages except for English, including French, Spanish, English & French, Dutch and Chinese. The distribution of languages is shown in Table 1. Some of bibliometric studies (29, 36-38) also revealed that the English language is the predominant language of publications in water field researches.

**Table 1:** Distribution of languages in journal articles

**Language**	**Total ** **publication (%)**	**Rank**
English	291(96.36)	1
French	5(1.66)	2
Spanish	2(0.66)	3
Chinese	1(0.33)	4
Dutch	1(0.33)	4
English; French	1(0.33)	4
Italian	1(0.33)	4

**Fig. 1:**
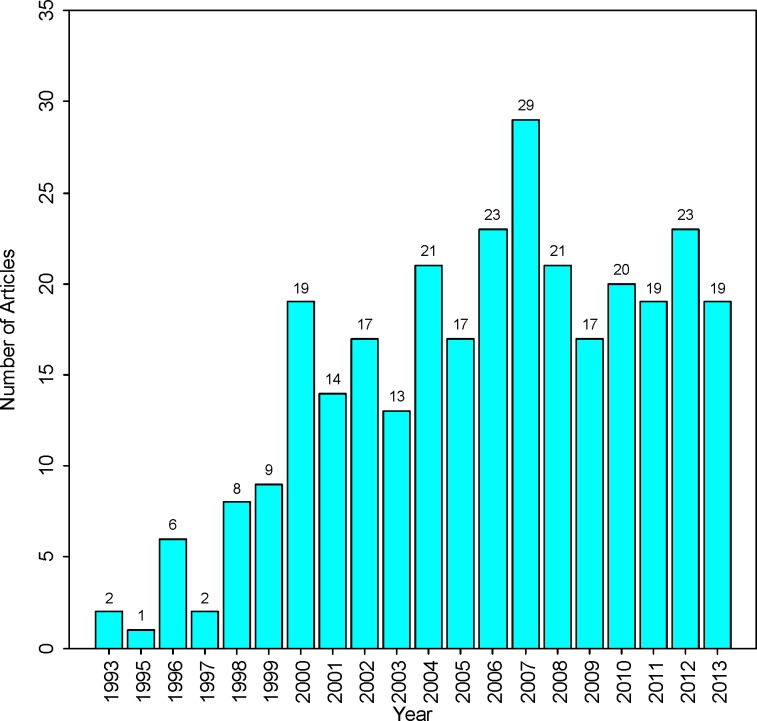
Annual journal articles publication

The distribution of annual publication output is shown in Fig. 1. There were an increasing number of publications mainly during a period from 1993 to 2007. From Fig. 1 it can be seen that the number of articles between 2006 and 2013 were not significantly changed. It may be predicted that a downward trend in the number of studies is likely to occur in future.

***Distribution of Countries***

The total article number for distribution analysis of country publications was 302. Among 302 articles, 219 (72.51%) were independent publications and 83 (27.48%) were internationally collaborative publications. The main productive countries were ranked by the number of total publications (Table 2). 

United States with 151 articles (50.0%), United Kingdom with 44 articles (14.57%), Australia with 36 articles (11.92%), Canada with 30 articles (9.93%) and Netherlands with 17 articles (5.63%) are the top five research countries. Furthermore, within these top productive countries, United States and Netherlands have the most (78.31%) and the least (6.02) internationally collaborated papers, respectively. 43.05% of United States’s articles and 29.41% of Netherlands’s articles are internationally collaborated publications.

**Table 2:** Productive countries in research on risk assessment of *Cryptosporidium *with at least 7 articles

**Country**	**SC(%)R**	**MC(%)R**	**TP(%)R**	**FA(%)R**	**CA(%)R**
United States	86(39.27)1	65(78.31)1	151(50)1	113(37.42)1	118(39.07)1
United Kingdom	28(12.79)2	16(19.28)2	44(14.57)2	36(11.92)2	38(12.58)2
Australia	21(9.59)3	15(18.07)3	36(11.92)3	28(9.27)3	26(8.61)3
Canada	21(9.59)3	9(10.84)4	30(9.93)4	26(8.61)4	25(8.28)4
Netherlands	12(5.48)5	5(6.02)7	17(5.63)5	15(4.97)5	13(4.3)5
France	8(3.65)6	8(9.64)5	16(5.3)6	11(3.64)6	13(4.3)5
Sweden	1(0.46)18	6(7.23)6	7(2.32)7	4(1.32)9	5(1.66)7

TP, Total publications; R, Rank; SC, Single country publications; MC, Multiple countries publications; FA, First author publications; CA, Corresponding author publications.

***Co-authorship relations among countries***

**Fig. 2:**
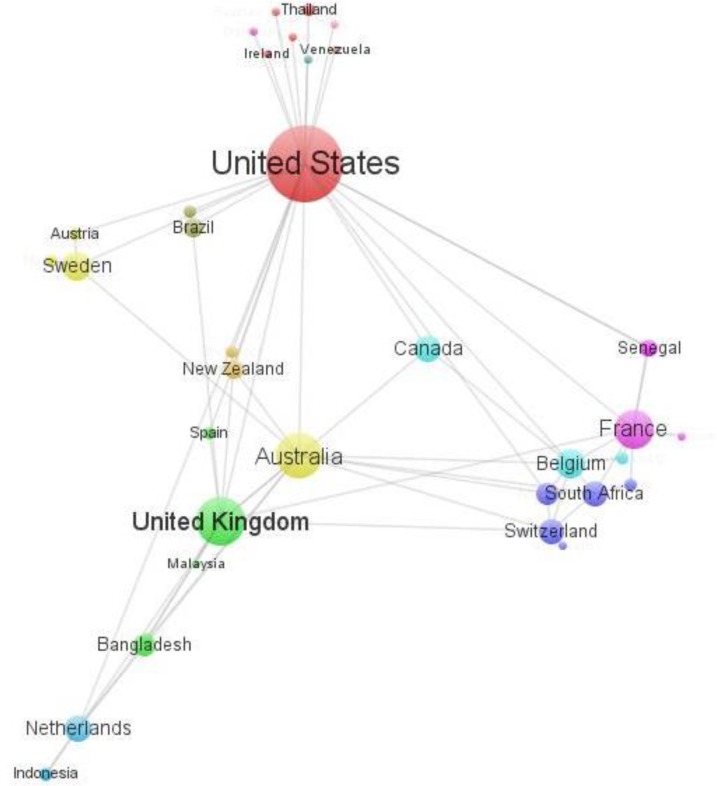
Bibliometric map based on the network of co-authorship relations among 36 countries

The bibliometric mapping is destined to evaluate academic outputs as publication and citation information of a particular field using statistical methods (39). The most common units of analysis in science mapping are journals, documents and authors (40). The relation among units can be represented as a graph or network, where the units are the nodes of circles and the relations among them represent a link between two nodes. In this study VOSviewer software (41) was used to create the bibliometric network maps for counties (Fig. 2) and authors (Fig. 4) relations.

Figure 2 shows the network among 36 countries of international co-authorship. In this view, countries are indicated by a label and by a circle. The more important a country, the larger its label and its circle. The size of each circle shows the number of papers written by authors from the country. Each link between two circles of different countries indicates that there is a co-authorship between the organizations in those countries. Out of the 84 co-authorship relations of countries, there are 3 top international co-authorship relations: Australia - United States (6.62%), Canada - United States (5.15%) and United Kingdom - United States (4.41%). There was the most co-authorship relations among European countries (20.69%) followed by European countries with United States (16.94%). There were also significant collaborations (11.03%) between the research institutions in the United States. The collaboration between Asian countries and United States is about one-third (5.9%) of that between European countries and United States. On the other hand, even with the considerable collaboration of Asian countries with other continents (12.55%), the number of research collaborations among Asian countries is zero.

Among the European countries, Netherland ranks first in articles production on the microbial risk assessment of *Cryptosporidium *in water field. Among the Asian countries, China ranks first with five articles publication and without international collaborations.

***Distribution of journals and subject categories***

Table 3 shows the distribution of output in journals. *Water Research*, *Journal of Water and Health*, *Water Science and Technology*, *Applied and Environmental Microbiology*, *Risk Analysis* and *Environmental Science and Technology* are the top 6 journals with more than 10 publications on the risk assessment of *Cryptosporidium *in water. Assessment of distributions of subject categories indicates that “environmental sciences” (238; 78.81%), “medicine” (118; 39.07%), “Immunology and Microbiology” (62; 20.53%) and “Engineering” (37; 12.25%) are the top 4 most popular subject categories.

***Distribution analysis of author keywords***

The assessment of author keywords revealed that from 554 author keywords, 432 (77.97%) keywords appeared only once. The large number of once-only author keywords probably indicated a lack of continuity in research and a wide disparity in research focuses (42).

**Table 3:** Distribution of the output Journals with at least 8 article publications

**Journals**	**TP (%)**	**Rank**	**IF**
Water Research	33(10.93)	1	4.655
Journal of Water and Health	31(10.26)	2	1.220
Water Science and Technology	29(9.6)	3	-
Applied and Environmental Microbiology	18(5.96)	4	3.678
Risk Analysis	12(3.97)	5	2.278
Environmental Science and Technology	10(3.31)	6	5.257
Epidemiology and Infection	8(2.65)	7	2.867
Science of the Total Environment	8(2.65)	7	3.258

TP, Total publication; IF, Impact Factor

Except for “*Cryptosporidium*”, “risk assessment” and “drinking water” which were searching keywords, two most frequently used keywords were “Giardia” (30; 9.93%) and “QMRA” (15; 4.97%). In most studies those the risk of *Cryptosporidium *were examined, the risk assessment of *Giardia *was also involved in the investigation. Therefore, this made “Giardia” as the most frequently used keyword in the research. The appearance of keywords “wastewater” (11; 3.64%) and “surface water” (8; 2.65%) reveals that the most importance sources of risk for these two pathogens may be wastewaters and surface waters. Furthermore, “Filtration” (6; 1.99%) and “disinfection” (5; 1.66%) which belong to treatment methods were also used. This is due the fact that after filtration as a treatment process the disinfection is the most effective method to inactivate the viable forms of *Cryptosporidium *and *Giardia *spp. 

***Distribution analysis of authors***

The distribution analysis of authors shows Ashbolt NJ form Canada is the most productive author in this field with 12 publications, which accounts about 3.97 % of the total publications. Two United states’ authors Rose JB (9; 2.98%) and Haas CN (7; 2.32%) ranked in the second and third positions with nine and seven publications, respectively. De Roda Husman AM (7; 2.32%) from Netherland also ranked in the third position with seven publications. These four top authors account about 11.59% of total publications.

***Authorship Pattern***

**Fig. 3:**
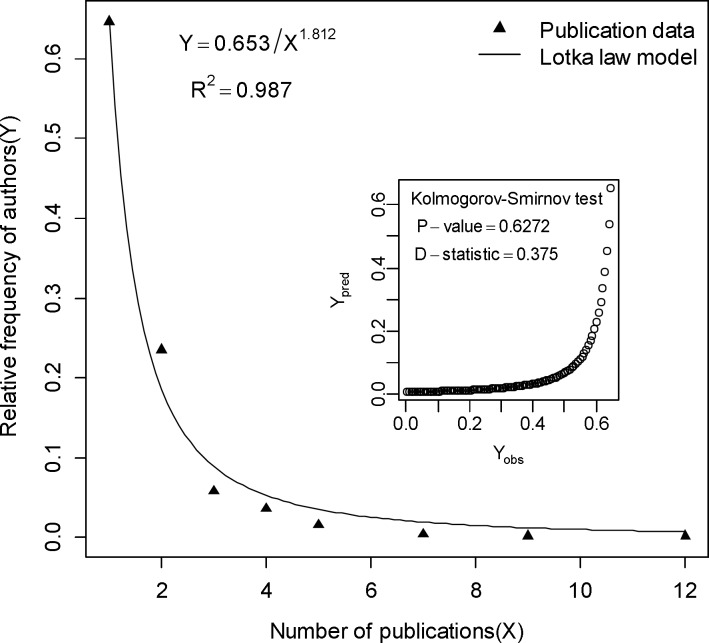
Relative frequency of authors versus the number publications

From the publication data, it has been revealed that 501 authors publish 302 articles, which is 0.60 articles per author. It means single authorship is common in this field. Fig. 3 shows the relationship between the relative frequency of authors and their publications. Lotka's law (43) describes the frequency of publication by authors in any given field. It states, “the number of authors making n contributions is about *1/n*^a^ of those making one; and the proportion of all contributors, that make a single contribution, is about 60 percent”. This means that out of all authors in a given subject field, about 60% publish only one article, 15% publish two articles, 7% publish three articles, and so on. According to Lotka's law only six percent of the authors in a given field will produce more than 10 articles. The generalized form of Lotka’s Law can be expressed as equation (1):


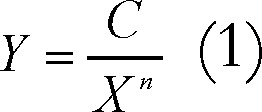


where *Y* is the relative frequency of authors with *X* articles, the exponent *n* and constant *C* are parameters to be estimated from a given set of author productivity data.

Using above formula and the modifications given by Pao and Fang (44-46) the value of *C* and *n* for the literature is determined to be 0.653 and 1.812, respectively. Kolmogorov-Smirnov Goodness-of-Fit test shows that literature does follow Lotka’s law (*P*=0.627). In this study the maximum (absolute) deviation is 0.375, leading to the acceptance of the Lotka’s law. 

***Co-authorship relations among authors***

**Fig. 4:**
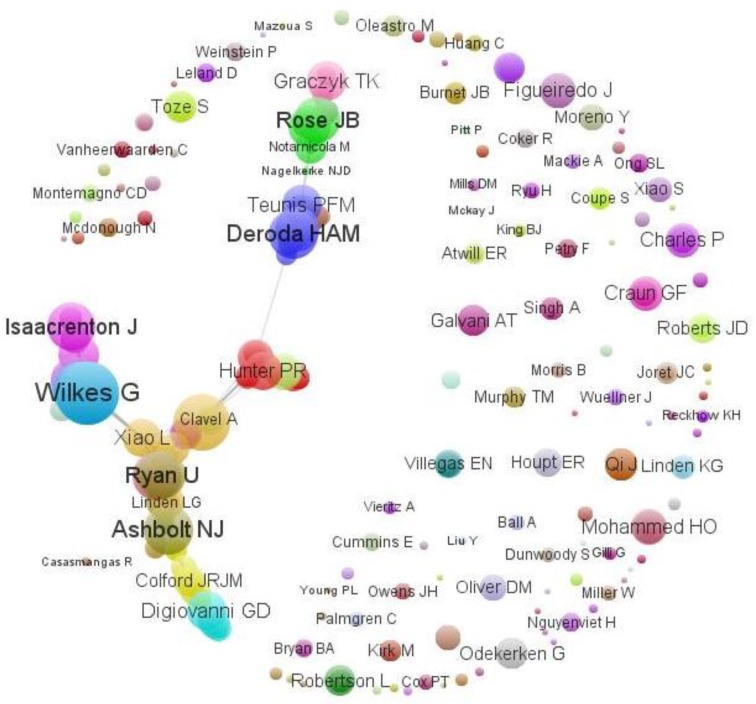
Bibliometric network map of co-occurrence of authors

Fig. 4 shows a co-occurrence network map generated from publications of the authors. Several different components including author nodes (circles), co-occurrence weight (circle size), networked relationship clustering (color and proximity), and name of authors (text) included in a map. Co-authorship relations are representing whether an author have written a paper with another author. Analyzing co-authorship information will assist in identifying groups of people who work closely together (47). The maximum number of the co-occurrences is attributed to four pairs of authors: Schets FM & Deroda HAM, Wade SE & Mohammed HO, Ruecker NJ & Neumann NF and Schijven JF & Deroda HAM, has been involved in the production of four articles (1.32 %).

In Fig. 4 the authors; Lapen DR, Topp E, Wilkes G similarly have the highest number of co-occurrences. Although these three authors have the greatest number of co-occurrence weights, each of them published only four articles, whereas Ashbolt NJ with 37 co-occurrence weights published 12 and Deroda HAM with 29 co-occurrence weights published 7 papers. Thus, these later two authors can be considered as the most influential authors in this field.

***Trends of research related to*** **Cryptosporidium pathogen**

***Cryptosporidiosis’s health effects and outbreaks***

*Cryptosporidium *was first described by E.E. Tyzzer in 1907 (48).There were no identified human cases until 1976 when Nimeet al. (49, 50) described cryptosporidiosis in a 3-year-old girl who was vomiting everything taken by mouth and had severe watery diarrhea. Since that time, *Cryptosporidium *pathogen has been recognized as a cause of gastrointestinal illness in both immune-competent (51) and immune-deficient people (52). In immune-competent people, *Cryptosporidium *leads to a self-limited illness, but in those who are immune-compromised, the infection can be unrelentingly fatal (53). Since 1976, there have been multiple outbreaks within the United States and United Kingdom. The most notable was the 1993 outbreak (54) in Milwaukee city in the U.S. state of Wisconsin where 403,000 infected. This massive outbreak had been caused by *Cryptosporidium *oocysts that were passed through the filtration system of one of Milwaukee’s water treatment plants (55). In United Kingdom, the notable outbreaks were 1989 outbreak in Swindon, 1990 outbreak in North Humberside (56) and 1995 outbreak in Torbay area of Devon (57). In response to the public concern the outbreaks of cryptosporidiosis, in 1990 a report of a group of experts from UK presented a comprehensive review of knowledge on *Cryptosporidium*, its occurrence in the environment and its importance as a waterborne infection for humans (56). These outbreaks in US and UK and Wisconsin especially, may have been the main trigger to initiate the investigations and a major reason that the number of total publications continuously increased from 1993 to 2007. During this period, the main research focus was how the likelihood of waterborne transmission can be decreased by identifying the approaches taken by countries (mainly UK, and US) government regulatory bodies (58).This may be the reason that a considerable proportion of published papers belongs to the United States and United Kingdom. Waterborne cryptosporidiosis outbreaks are more common than outbreaks involving other means of transport (59). About 199 outbreaks of human diseases due to the waterborne transmission of parasitic protozoa were occurred and reported during the period from 2004 to 2010. 46.7% of the reported outbreaks were occurred on the Australian continent, 30.6% in North America and 16.5% in Europe. Cryptosporidium spp. was the etiological agent in 60.3% of the outbreaks and Giardia Lambliain 35.2% (59). These may be the reasons that Australia was ranked third country in producing the article publications and “Giardia” was frequently used author keyword. Most commonly *Cryptosporidium *species associated with human cryptosporidiosis are *C. parvum*, *C. hominis* and *C. cuniculus* (60). Analysis of author keywords reveals the *C. parvum *is further considered species in comparison with others.

***Quantifying the risk of*** **Cryptosporidium**

Quantifying the risk of *Cryptosporidium *can be done through a QMRA study. The QMRA is an approach that brings information and data together with mathematical models to deal with the spread of microbial agents through environmental exposures and to describe the nature of the adverse outcomes (61). The QMRA has four stages, based on the national academy of sciences framework (62) for quantifying the risk, but this framework was modified to account for the properties of living organisms. These four stages include *Hazard Identification, which describes a microorganism,* and the disease it causes, including symptoms, severity, and death rates from the pathogen. The relationship between the dose (number of pathogens) received and the resulting health effects described in *Dose-Response *stage. Data sets from human and animal studies allow the construction of mathematical models to predict dose-response. Examples of mathematical dose-response relationships for *Cryptosporidium *include an exponential model with an ID_50_ of 165 oocysts developed by DuPont et al. (63). An exponential model and a Beta-Poisson model were also provided by Messner et al. (64). The exponential model by Messner et al. is preferred in most circumstances (65). However, all available models should be considered to decide which one is most appropriate for analysis. In *Exposure Assessment* stage the pathways that allow a pathogen to reach people and cause infection is described. The size and duration of exposure by each pathway, the number of people exposed and the categories of people affected are determined in this stage. Information of previous stages is integrates into a single mathematical model to calculate risk - the probability of an outcome - in *Risk Characterization* stage. Since previous stages will not provide a single value, but a range of values for hazard, dose and exposure, thus the risk needs to be calculated for all values across those ranges. Monte-Carlo analysis is required in this case, and the result is a full range of possible risks, including average and worst-case scenarios. 

***Determination*** ***of*** **Cryptosporidium in** ***drinking water***

In response to the risk of *Cryptosporidium *to public health, the EPA developed draft Method 1622 for *Cryptosporidium *detection in December 1996. *Cryptosporidium*-only method was validated in 1998, and was revised again as a valid method for detecting *Cryptosporidium *in water in 1999 (66). In the same year, EPA validated a new method for simultaneous detection of *Cryptosporidium *and Giardia. To avoid confusion with Method 1622 as a stand-alone *Cryptosporidium*-only detection method, EPA designated the new combined procedure EPA Method 1623 (67). Both methods were revised in 2001, 2003 and 2005. Approval of the use of portable continuous-flow centrifugation was included in the 2005 modified version of the methods (68). Method 1623 was again revised in 2012 to become Method 1623.1 (69). This revised method is for the detection of *Cryptosporidium *and *Giardia *in water by concentration, immunomagnetic separation (IMS), and immunofluorescence assay (FA) microscopy. In method 1623.1 *Cryptosporidium *and *Giardia *are further characterized using 4', 6-diamidino-2-phenylindole (DAPI) staining and differential interference contrast (DIC) microscopy. This method identifies the genera, *Cryptosporidium *or *Giardia*, but not the species and cannot determine the host species of origin, nor can it determine the viability or infectivity of detected oocysts and cysts (69). The DNA extraction followed by PCR amplification, PCR sequencing and computer database homology comparison (CDHC) were also recently used as a method to screen various water sources for public consumption for the presence of *C.* *parvum*, *Cyclospora cayetanesis*, and *G. lamblia* (70). Development of detection methods and modifications of these methods up to 2005 may be one of the reasons, which encouraged the researchers to doing further investigation from 1993 up to 2007.

***Removal of*** **Cryptosporidium** ***from drinking water***

Cryptosporidial infection is transmitted through fecal-oral route by contaminated water and food (71). Waterborne cryptosporidiosis outbreaks are more common than outbreaks involving other means of transport. Up to the end of 2010, 185 outbreaks had been reported globally (59) contrasted with less than 20 foodborne outbreaks (72). This is only partly because of the features of *Cryptosporidium *favoring waterborne transmission (60). The oocyst of *Cryptosporidium *species is highly resistant to common water disinfection processes and can remain infectious for prolonged periods in the environment (73). For preventing human exposure, oocysts must be removed from water supplies. Inadequate water filtration can increase persons expose to risk for infection from viable oocysts (74). Systems using surface water or ground water under the direct influence of surface water must disinfect and filter the water, so that 99 percent of *Cryptosporidium *oocysts (2-log of removal) are removed or inactivated (75). Unfiltered systems that meet criteria for avoiding filtration are required to include *Cryptosporidium *in their existing watershed control provisions (75). The water treatment plants generally have to provide a level of treatment consistent with at least 2-log *Cryptosporidium parvum*, 3-log *Giardia *lamblia, and 4-log virus reduction on a continuous basis, regardless of the actual measured treated water quality (76). Of the technologies available to the drinking water industry, coagulation followed by micro-filtration (> 6-log removal) (77), ultra-filtration (> 6-log removal) (77), diatomaceous earth filtration (>4-log removal) (78) and slow sand filtration (>3.7-log removal) (78) appear to provide the most levels of *Cryptosporidium *removal. Conventional treatment systems appear capable of meeting 2-log removals in most of the cases studied (79). These make “disinfection” and “filtration” as frequently used keywords in publications.

## Conclusion

The historical review and contributions on the characteristics of the *Cryptosporidium*-related risk assessment research activities were assessed by bibliometric methods in this study. The study indicates that there are an increasing number of annual publications mainly from 1993 to 2007. This increase was mainly due to successive outbreaks of cryptosporidiosis in countries such as United States and United Kingdom. The public health threats of outbreaks and the necessity for finding the postulated their reasons toward prevention of the outbreaks, were main reasons, which caused an increase in the number of annual publications in this period. 
